# Synergistic Effects of Magnesium Oxide and SBR Latex Additives on Cement Sheath Stability in Oil Well Operations

**DOI:** 10.1021/acsomega.4c05811

**Published:** 2024-08-15

**Authors:** Ramón V. A. Ramalho, Salete M. Alves, Júlio C. O. Freitas, Willame G. S. Batista, Fabricio P. F. Silva

**Affiliations:** † Mechanical Coordination, Federal Institute of Education, Science and Technology of Alagoas, 57230-000 Coruripe, Alagoas, Brazil; ‡ Oil Well Cementing Technology Center, Institute of Chemistry, Federal University of Rio Grande do Norte, 59078-900 Natal, Rio Grande do Norte, Brazil; § School of Science and Technology, Federal University of Rio Grande do Norte, 59078-900 Natal, Rio Grande do Norte, Brazil

## Abstract

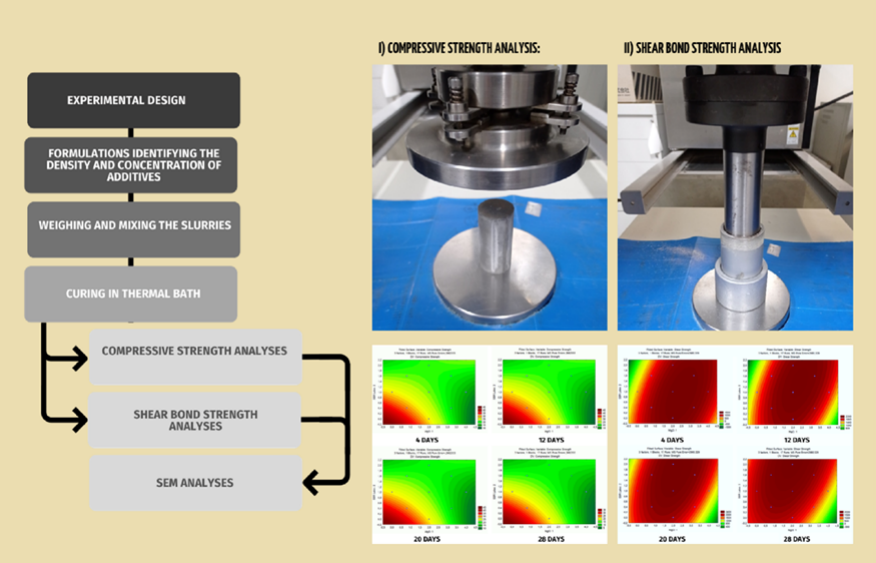

Leaks through cement sheaths remain a complex and challenging issue in the oil industry, representing a persistent obstacle that has endured for decades. The drying shrinkage, an inherent characteristic of Portland cement, substantially exacerbates this problem, driving the formation of microcracks and heightened permeability under variable stress conditions. In this context, additives emerge as significant elements in addressing this issue, offering a pathway to mitigate the adverse effects of leaks. Among these additives, magnesium oxide (MgO) stands out for its ability to reduce drying shrinkage through structural modifications in the cement matrix. Simultaneously, SBR Latex, another important additive, acts to minimize gas migration due to its polymeric microstructure while also strengthening acid resistance and enhancing microstructural cohesion. This study aims to deepen the understanding of the interaction between MgO and SBR Latex additives in cement slurries, employing an experimental design to substantiate and expand upon the analyses conducted. The results reveal a synergistic integration of these additives, with MgO acting as an effective agent in reducing drying shrinkage and gel formation, thereby contributing to the strengthening of shear strength. Conversely, SBR Latex provides elasticity to the slurry, although with a slight compromise in compressive strength, with a relatively limited effect on shear strength. The strategic combination of these additives results in improvements in the mechanical integrity of cement slurries, a positive advancement in the context of petroleum well cementing operations. Thus, this study not only highlights the individual properties of MgO and SBR Latex but also offers valuable perspectives for the careful formulation of cements, with potential applications in challenging operational environments in the oil industry.

## 1 Introduction

Leakage through the cement sheath has been a constant problem over the years.^1^ This leakage can turn oil wells into sources of effluents that pollute both underground and surface regions.^1^ Even after a successful cementing job, the cement itself can degrade over time due to mechanical, chemical, or combined processes. Mechanical degradation stems from factors like excessive load stress on the cement sheath, thermal expansion, and volume changes during hydration.^2^ On the other hand, chemical degradation can arise from physical-chemical reactions caused by exposure to acidic fluids.^3^

Extensive research over the years has focused on mitigating damage from failures in the cementation process, leading to the incorporation of several scientific discoveries into professional field practices. Consequently, various additives have been employed to enhance the cement matrix and improve its adaptability to well conditions.

Magnesium Oxide (MgO) stands out as a crucial additive recognized for its expansive properties and its ability to reduce porosity during cement hydration.^4^ These characteristics are attributed to the formation of Mg(OH)_2_ within the pore spaces of the cementitious matrix during the hydration process.^4^ MgO has also demonstrated a significant influence on other cement slurry properties, including compressive strength^5−8^ and shear strength.^9,10^ Furthermore, specialized cement slurries incorporating MgO have been developed to address specific challenges in well cementing, particularly in salt formations. These slurries exhibit enhanced properties such as high early strength and low permeability, crucial for preventing gas migration and ensuring long-term well integrity.^11^

Similarly, SBR Latex is widely used as an additive in cement slurries for both oil wells and civil construction. Its widespread use stems from its ability to prevent gas migration by reducing fluid loss, increasing acid resistance, and improving high-temperature properties. Latex also enhances mechanical properties by increasing the slurry’s deformation capacity, minimizing communication between zones, and increasing viscosity. As polymeric additives, latices improve flexibility and reduce the inherent fragility of ceramic composite materials like cement.^12−14^

Numerous studies have investigated the performance of latex as a cement slurry additive in oil wells, revealing its significant impact on slurry properties. Latex has been observed to improve compressive strength and enhance adhesion to the wellbore surface.^15^ Research highlights various beneficial effects of latex additives on cement slurry properties. For instance, latex retards the hydration process, improving workability and reducing bleeding.^16^ Studies have shown that SBR latex enhances workability, strength, and adhesion of cement slurries,^17^ while also boosting compressive strength, durability, and abrasion resistance.^18^ Furthermore, latex improves suspension stability, reduces segregation, and increases both compressive strength and adhesion.^19^ In the context of oil wells, SBR latex significantly influences the hydration kinetics of cement slurries, impacting the overall performance of well cementing.^20^ It also modifies the microstructure of cement slurries, promoting a more uniform pore distribution and increasing mechanical strength.^21^ The addition of SBR latex significantly alters the rheological properties of Class G Portland cement slurries with silica fume.^22^ Despite these findings, the use of latex in cement slurries, similar to the application of MgO, has not been extensively discussed in the literature.

The scientific analysis of SBR Latex behavior often takes place within the industry, with several service companies employing latices as additives to control fluid loss and gas migration. This control over gas migration contributes to improved cement adhesion to the rock formation and metallic coating.^23^ Studies have explored multicomponent cements containing latex to enhance the sealing performance of casing columns in boreholes. These cements exhibit improved properties, including reduced permeability, increased bonding strength, and enhanced resistance to aggressive fluids, contributing to the long-term integrity of wellbore seals.^24^

In the context of oil wells, both additives, MgO and SBR Latex, contribute to reducing contamination caused by leaks by controlling crack formation.^24^ However, they operate through different mechanisms, and there is a lack of research on their combined effects within the cementitious matrix.

Optimizing spacer pumping time is crucial for efficient cementing operations.^25^ Minimizing the transition time between drilling mud and cement slurry reduces the risk of contamination and subsequent cement sheath degradation, improving well integrity.^26^ Additionally, understanding the influence of fine-grained materials, on cement slurry parameters is essential for tailoring slurry properties to specific well conditions.^27^ These materials can impact properties like density, viscosity, and filtration, ultimately affecting the success of cementing operations.^27^ Furthermore, the migration of natural gas in boreholes poses significant risks to well integrity and safety.^28^ Laboratory studies have focused on understanding the mechanisms of gas migration and developing effective mitigation strategies to limit gas migration, minimizing the potential for cement sheath damage and environmental contamination.^28^

Building upon this background, this work investigates the individual and combined effects of MgO and SBR Latex additives on the compressive and shear strengths of cement matrices. To optimize this process, all analyses were conducted using experimental designs, providing valuable statistical insights for evaluating the results. This approach allows for the identification of optimal scenarios and the development of projections for different contexts.

## 2 Methodology

The present study analyzed properties of cement slurries varying the SBR latex additive concentrations between 0 and 2 gal/ft^3^, magnesium oxide between 0% and 4% and curing times (4 and 28 days). Pressure and temperature values usual in oil wells in the northeast region of Brazil were used to determine the slurry formulations used in this work. The fixed well depth was 760 m, with bottom hole circulating temperature (BHCT) of 93 °F (34 °C) and bottom hole static temperature (BHST) of 118 °F (48 °C), based on a geothermal gradient of 1.5 °F/100 ft.

### 2.1 Design of Experiments

A Central Composite Rotatable Design (CCRD) 2^3^, including 6 axial points and 3 repetitions at the central point, totaling 17 trials. For the 2^3^ factorial design, three analysis variables were established: MgO concentration (% bwoc, by weight of cement), latex concentration (gpc, gallon of additive per cubic foot of cement slurry), and curing time (days). The variation of these factors was analyzed as a function of the responses of the slurries in the Compressive Strength Analyses (CSA) and Shear Bond Strength Analyses (SBSA).

The variable MgO concentration was analyzed from 0 to 4%, widely used in the literature. Values above this usually do not show a significant change in the results.^29^

SBR Latex is an additive widely used in the composition of cement slurries for oil wells, capable of helping to contain the passage of gas through the cement sheath and generating flexible behavior in the slurries. The SBR Latex Concentration variable was analyzed from 0 to 2 gpc (0 to 267.5 L of additive per m^3^ of cement slurry). This interval is sufficient because, considering the analyses present in the literature, concentrations greater than 2 gpc do not have a relevant effect and can also cause adverse effects such as loss of miscibility of the slurry, that is, the inability of the cement slurry to mix adequately with the well fluids.^30−32^

The curing time was varied between 4 and 28 days. This variable is examined in cementing works as it adjusts the setting speed and regulates the properties of the slurry according to the needs of each well. In the course of cement curing, hydration stages are fulfilled and promote the hardening of the slurry. When exposed to mechanical efforts, even in critical stages of the hardening process, the slurry may respond insufficiently, which should be avoided.

Table 1 describes the combinations of variables used and the variation intervals established by a Central Composite Rotatable Design (CCRD):

**Table 1:** Values Used in the CCRD for Three Factors

	–1.68	–1	0	1	1.68
MgO	0	0.8	2	3.2	4
SBR latex	0	0.4	1	1.6	2
curing time	4	9	16	23	28

These variations are combinations determined through the statistical method present in the experimental design. The combinations between factorial points (1 and −1) represent the first eight tests. The following six tests combine an axial point (1.68 or −1.68) with the center point value (0). Finally, there are three more repeating points with the center point values of the three variables involved.

From the computationally analyzed CCRD data, the response surface graphs were obtained by fitting a mathematical model to the collected data, enabling the visualization of interactions among the studied variables. These graphs provided a three-dimensional representation of the relationship between the independent variables and the process response, allowing for the identification of optimization regions. Pareto diagrams were generated to highlight which factors had the greatest impact on the process response, ranking the bars in descending order of magnitude. Furthermore, critical values were calculated, indicating the optimal points for the controlled variables, maximizing or minimizing the desired response. These analyses provided a deeper understanding of the process and guided decision-making for optimization and improvement.

Through analysis of variance, it is possible to identify a statistically reliable regression model capable of determining predictive response values through a statistical framework. In this manner, quadratic equations were established with the concentrations of additives and curing time for the model. The experimentally obtained data were fitted to a second-order model, eq 1:
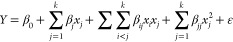
1

In this context, *Y* stands for the measured mechanical property, and β_0_ represents the mean data set. The quadratic and interaction terms are symbolized as β*_j_*, β*_jj_*, and β*_ij_*, respectively. The variables *x*_*i*_ and *x*_*j*_ indicate the values of the independent variables, and ε denotes the normal distribution of random errors. The findings underwent analysis with statistical software capable of producing response surface plots derived from the second-order model equation. The accuracy of this equation was gauged using the coefficient of determination *R*^2^, while its statistical significance was assessed via the *F*-test (analysis of variance).

Table 2 presents the 17 combinations of this factorial design, with the amounts used in the formulations.

**Table 2:** Non-Coded Values for the Components of the Formulations

	MgO (*x*_1_)	SBR latex (*x*_2_)	curing time (*x*_3_)
	%	gpc	days
1	0.8	0.4	9
2	0.8	0.4	23
3	0.8	1.6	9
4	0.8	1.6	23
5	3.2	0.4	9
6	3.2	0.4	23
7	3.2	1.6	9
8	3.2	1.6	23
9	0	1	16
10	4	1	16
11	2	0	16
12	2	2	16
13	2	1	4
14	2	1	28
15 (C)	2	1	16
16 (C)	2	1	16
17 (C)	2	1	16

### 2.2 Materials

The preparation of cement slurries on a laboratory scale maintained the same criteria executed in the field. Each slurry was prepared with a total volume of 600 mL, and this procedure is standardized using the API RP10B standard.^33^

In all slurries prepared for this work, the usual components, cement and water, and the additives Defoamer, Dispersant, Filtrate Control, Magnesium Oxide, and SBR Latex were used, as described in Table 3.

**Table 3:** Components Used in Formulations

components	description	specific volume (gal/lb)
cement	Class G Portland Cement	0.0382
water	potable water	0.1202
defoamer	silicone-based antifoam additive	0.1223
dispersant	dispersant additive based on modified lignosulfonate	0.1089
filtrate control	cellulose-derived filtrate control additive	0.1997
magnesium oxide	white hygroscopic solid mineral	0.0397
SBR latex	anionic dispersion of a noncarboxylated butadiene-styrene copolymer	0.1163

Portland Cement used for well cementing was supplied by Mizu S/A and nondistilled drinking water. The defoamer, dispersant, and filtrate control additives were used at concentrations of 0.03 gal/ft^3^, 0.035 gal/ft^3^, and 0.3% BWOC respectively. These concentrations were used fixedly for all formulations to adjust the properties as they are usual for cement slurries. The Magnesium Oxide (MgO) additive and the SBR Latex additive were the subjects of study, and their concentrations varied throughout the analyses in different scenarios.

### 2.3 Samples Preparation

Two mixtures were initially produced while mixing the components and preparing the slurries: the dry blend and the mixing water. The dry blend contained cement and Magnesium Oxide, whereas the mixing water included SBR Latex, a dispersant, defoamer, and filtrate controlling additive.

The tests were carried out using slurries in a cured state, and the curing times used throughout the tests varied from 4 to 28 days. The specimens remained in the Nova Ética Model 500/3DE Thermostatic Bath, simulating a bottom hole circulating temperature (BHCT) of 48 °C.

### 2.4 Uniaxial Compressive Test

After mixing, the prepared slurry was poured into three cylindrical molds measuring 38 mm in diameter, made of materials that are inert to chemical attack by cement.

The molds filled with cement slurry were sealed with plastic film and sent to the thermostatic bath. The thermostatic bath has dimensions suitable for complete immersion of the molds and also a water circulation system carried out by an agitator in order to guarantee a uniform temperature throughout the volume: the selected curing temperature, 48 °C.

After 4, 9, 16, 23, or 28 days submerged in the thermostatic bath, the molds were removed from the bath and demolded. The compressive strength was measured on a Shimadzu Autograph Model AG-I Universal Testing Machine controlled by the TRAPEZIUM 2 program.

### 2.5 Shear Bond Strength Test

The bonding of cement to the metal coating and rock formation is typically reported through the concept of adhesion. The force required to initiate movement of the casing in the cement sheath or movement of the formation cement, is defined as the shear strength. The rupture caused by this force involves damaging the connection between the coating and the cement or between the cement and the formation, preventing fluid flow.

The analysis of Shear Bond Strength (SBSA) is fundamental in oil well cementing. It assesses the effectiveness of the bond between the well casing, cement, and geological formations. SBSA allows for the identification of failure areas and ensures the structural integrity of the well, preventing leaks and other operational issues. This contributes to operational safety and the efficiency of oil and gas production, reducing risks and maintenance costs.

The Shear Bond Strength analysis was carried out through tests developed following the guidelines proposed in the document Guidelines on Qualification of Materials for the Abandonment of Wells^34^ developed by Oil & Gas UK, the leading trade association for the offshore oil and gas industry from the UK.

This test makes it possible to establish the value of the force necessary to break the bond between the cement matrix and the metallic coating. From this value and considering the contact area between the two parts, it is possible to calculate the shear resistance that each formulation presents at different values of the variables. In other words, this analysis allows us to evaluate how much the concentration of MgO and SBR Latex and the curing time can impact the shear strength of cement slurries applied to oil wells.

**Figure 1:**
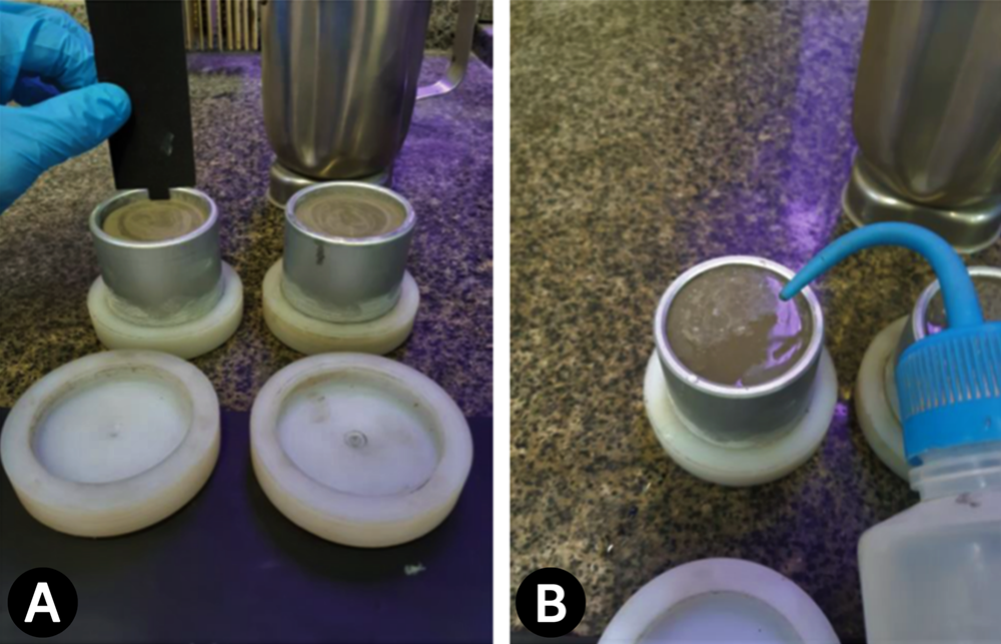
(A) Filling the cell, leaving a 5 mm space at the top; (B) filling the headspace with water.

**Figure 2:**
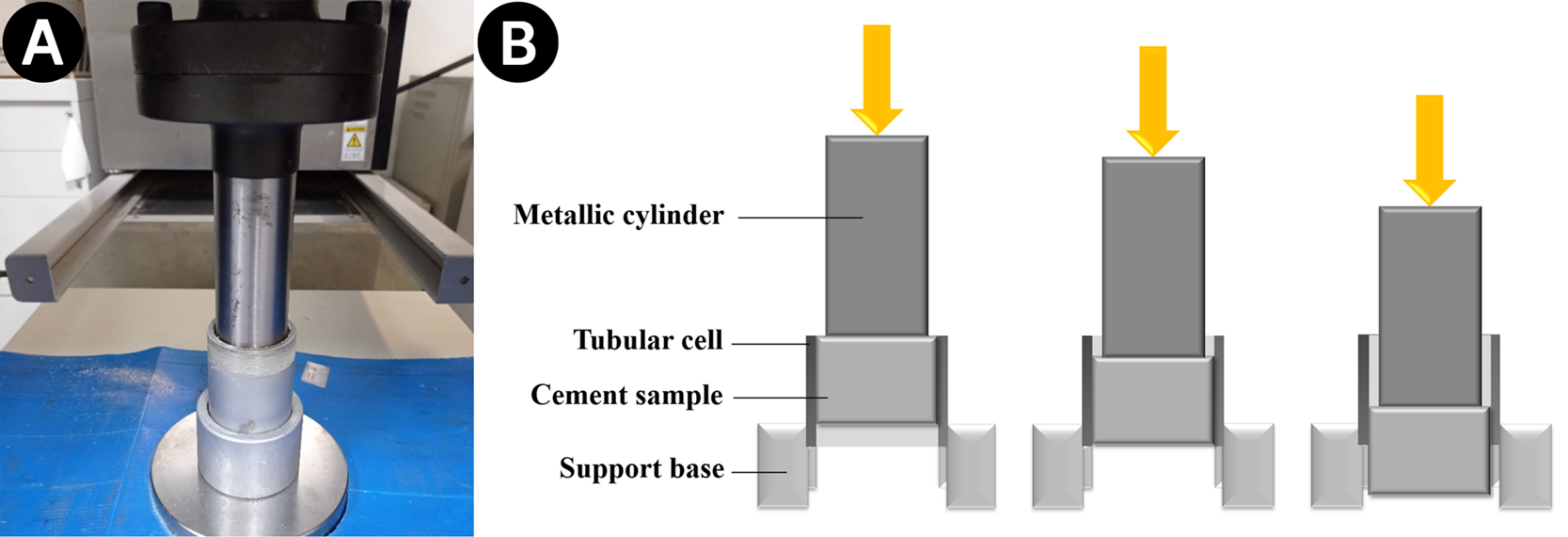
(A) Cell positioned and apparatus assembled to carry out the Shear Bond Strength Test; (B) illustrative steps of movement during the Shear Bond Strength Test.

The appropriate shear bond strength to the analysis conditions of this work follows the following steps:

1. The cement slurry is prepared and poured into the cell, leaving a space of 5 mm at the top, as shown in Figure 1A. A nylon cap is used at the lower end of the cell to support the material;
2. The cell is evacuated of air to remove bubbles in the material, and the head space is filled with water, Figure 1B. The cell is placed in a thermostatic bath for curing for a period of 4, 9, 16, 23, or 28 days according to the experimental plan;
3. After curing, the nylon cover is removed, and the cell is inverted to align the flat surface in contact with the steel cylinder used to apply load to the test assembly, as shown in Figure 2A. The assembly is placed inside a load cell. The steel rod must be centered in relation to the sides of the tubular cell.
4. The cell is loaded from the top through the solid steel bar at a rate of 13 mm/min (0.5 in/min) until the bond between the material and the tubular cell fails. The development of the assay is depicted in Figure 2B.
5. The shear strength (τ_b_, kN/m^2^) is obtained from the equation (eq 2):
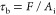
2where: *F* = the force required to break the bond (kN); and *A*_i_ = area of the inner surface of the tubular cell in contact with the material (m^2^).

### 2.6 Scanning Electron Microscope (SEM)

The Scanning Electron Microscope (SEM) was employed in the analysis of the microstructure of cement slurries. SEM is a high-resolution technique widely recognized for its capability to investigate the morphology and structure of materials at microscopic scales. In this study, SEM images were acquired to examine the characteristics of the microstructure of cement slurries with different concentrations of SBR Latex and MgO. This approach allowed for a detailed analysis of the interactions between the components of the mixture and their influence on the microstructure of the cement samples.

## 3 Results and Discussion

Table 4 describes the experimental planning and the values obtained for the response variables compressive strength and shear strength.

**Table 4:** Experimental Results of Compressive Strength and Shear Strength Analysis

	MgO (*x*_1_)	SBR latex (*x*_2_)	curing time (*x*_3_)	compressive strength	shear strength
	%	gpc	days	MPa	kN/m^2^
1	0.8	0.4	9	31.59	1707.86
2	0.8	0.4	23	27.21	2148.44
3	0.8	1.6	9	23.24	1472.43
4	0.8	1.6	23	16.69	2084.48
5	3.2	0.4	9	20.70	1873.25
6	3.2	0.4	23	14.51	1592.08
7	3.2	1.6	9	21.02	2004.12
8	3.2	1.6	23	15.76	2220.89
9	0	1	16	28.83	1740.68
10	4	1	16	14.04	1655.98
11	2	0	16	27.90	2156.80
12	2	2	16	19.98	2458.33
13	2	1	4	20.3	1576.47
14	2	1	28	15.86	1934.35
15 (C)	2	1	16	22.58	2512.38
16 (C)	2	1	16	21.60	2454.82
17 (C)	2	1	16	22.47	2403.25

**Figure 3:**
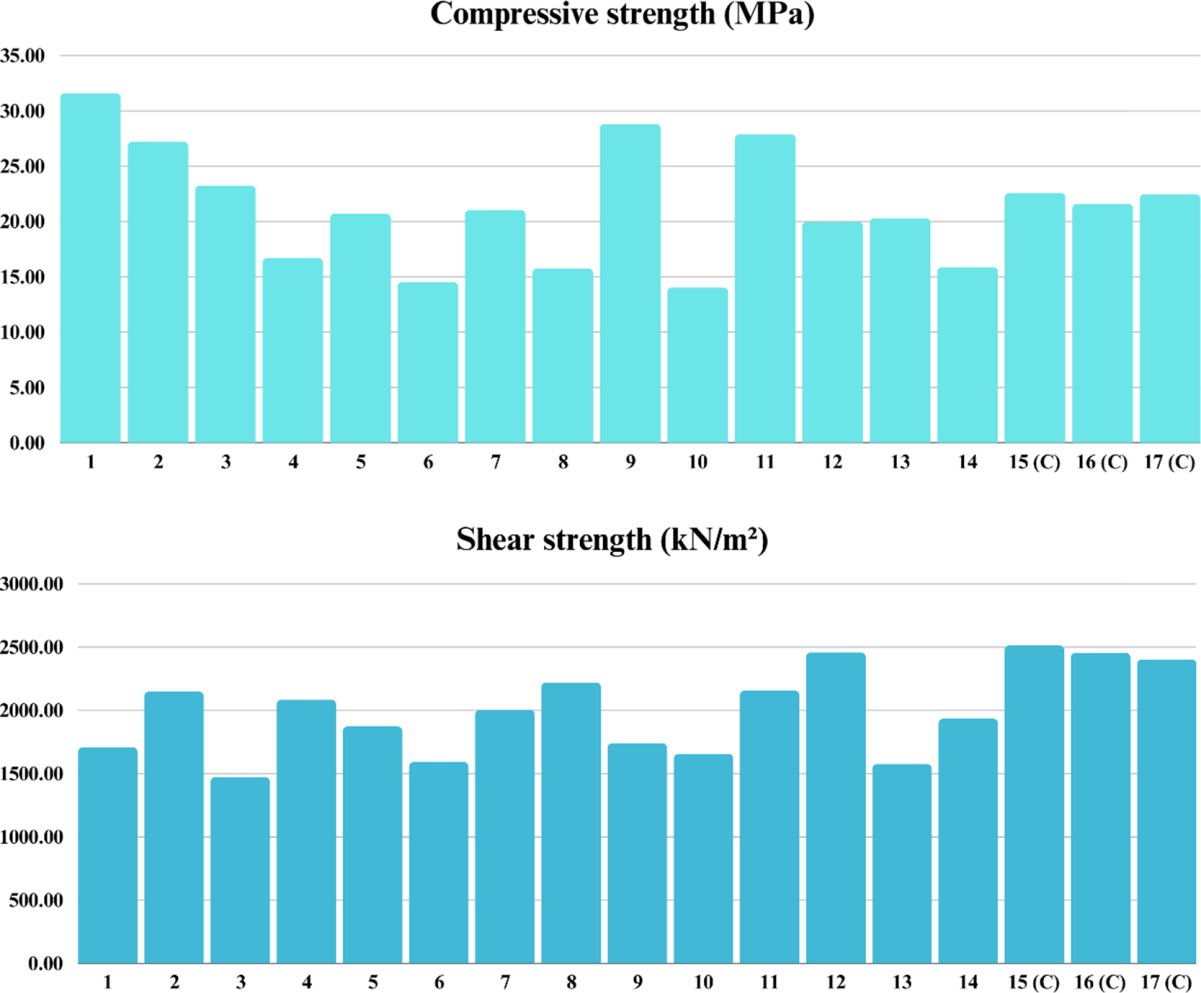
Graphical representation of the response variables compressive strength and shear strength.

Figure 3 graphically depicts the data from Table 4 regarding the response variables compressive strength and shear strength.

### 3.1 Compressive Strength Analyses (CSA)

#### 3.1.1 Quadratic Model Equation

The CSA results were subjected to the multivariate regression process, which aims to describe the relationships between the explanatory variables of the experiments. The quadratic regression showed a better fit with the obtained results.

The quadratic model equation is obtained through the quadratic regression process, which, based on the coefficients of the estimated effects of the factors that presented statistical significance (in red), the parabola equation that best fits the set of data provided is obtained.

The quadratic model equation was derived through a quadratic regression process. This process involves analyzing the coefficients of the estimated effects of the factors that have been found to be statistically significant, herein indicated in red. A parabolic equation was generated from these coefficients that best fit the data set under investigation. This equation describes the relationship between the variables and makes predictions or analyzes the impact of different factors on the observed outcomes.

Quadratic regression is a way of modeling a relationship between a set of variables. The result is a regression equation that can be used to predict the data.

In a table of regression coefficients, the symbols ″(*L*)″ and ″(*Q*)″ indicate the form in which the variables are used in the model. ″(*L*)″ refers to a variable included in a linear form, while ″(*Q*)″ indicates that the variable is included in a quadratic form. That is, *X*(*L*) represents the variable *X* without transformation, and *X*(*Q*) represents *X*^2^, the value of *X* squared.

Considering the data in Table 5, the quadratic model equation (eq 3) below was obtained, representing the compressive strength response of cement slurry systems resulting from the variation in the MgO and SBR Latex concentration and the curing time.
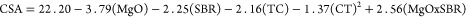
3

**Table 5:**
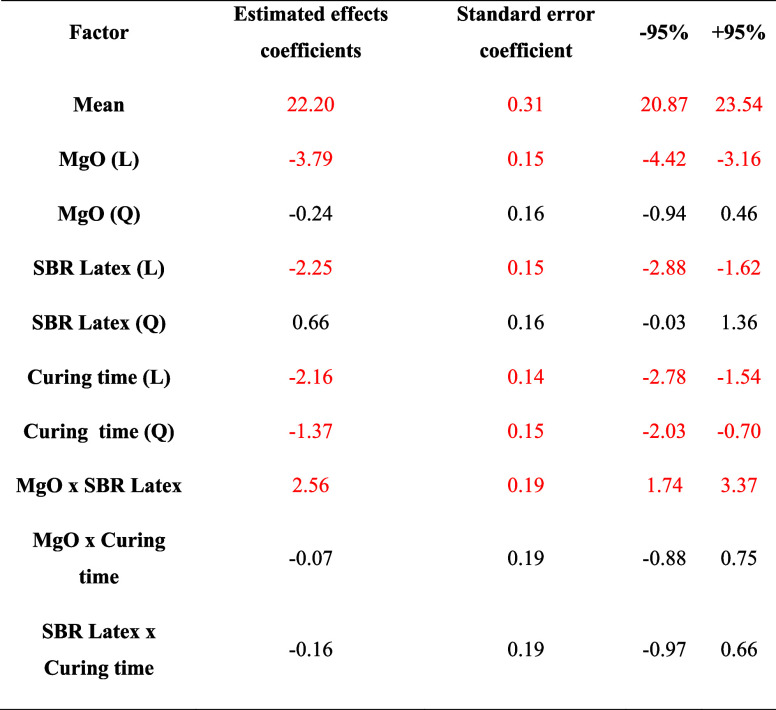
Compressive Strength Analysis Regression Coefficients

#### 3.1.2 Analysis of Variance: ANOVA

To define a model as highly significant, it must be tested through several factors. Some of these tests can be conducted using analysis of variance (ANOVA), such as the sequential F-test, and the results are presented in Table 6.

**Table 6:** ANOVA for the Quadratic Model^a^

source of variation	sum of square	degrees of freedom	mean square	*F* test
regression	417.56	9	46.40	23.84
residual	13.62	7	1.95	
lack of fit	13.04	5	2.61	9.05
pure error	0.58	2	0.29	
total	431.18	16		

a *F*_0.05;9;7_ (*F*_table_) = 9.05.

Therefore, it is necessary to obtain an *F*-test value (23.84) greater than the tabulated *F* value (9.05) to ensure the viability of the model equation. This requirement was met, as the *F*-test value was 2.63 times greater than the tabulated *F* value. The tabulated *F* value was determined using the *F* distribution table for a 95% confidence interval.

Another coefficient capable of attributing significance to the model is the coefficient of determination (*R*^2^), which provides a measure of the proportion of variation explained by the regression equation relative to the variation in the response. Generally, *R*^2^ is expressed in decimal terms, indicating how well the model equation fits the observed responses. The *R*^2^ value is determined by the ratio of the sum of squares for regression to the total sum of squares. A perfect model fit occurs when *R*^2^ is equal to 1, which only happens if there is no residual error and all the variation is due to the regression, which is unlikely. In this study, the *R*^2^ was 0.968.

Even for an appropriate model, it is also necessary to obtain a regression *F*-test value (23.84) greater than the lack-of-fit *F*-test value (9.05). This comparison legitimizes the viability of the model.

#### 3.1.3 Observed and Predicted Values

The obtained equation (eq 3) is evaluated as a well-representative equation of the analyzed system when the observed values are grouped close to the predicted values.^35^ A graph of predicted values vs observed values demonstrates how close the predictions from quadratic regression are to the values observed in the laboratory. Another graph that contributes significantly to the acceptance of the model is the Pareto diagram, which demonstrates what variables present significant interaction effects for the constituted model, that is, which variables can impact the result of the dependent variable compressive strength.

**Figure 4:**
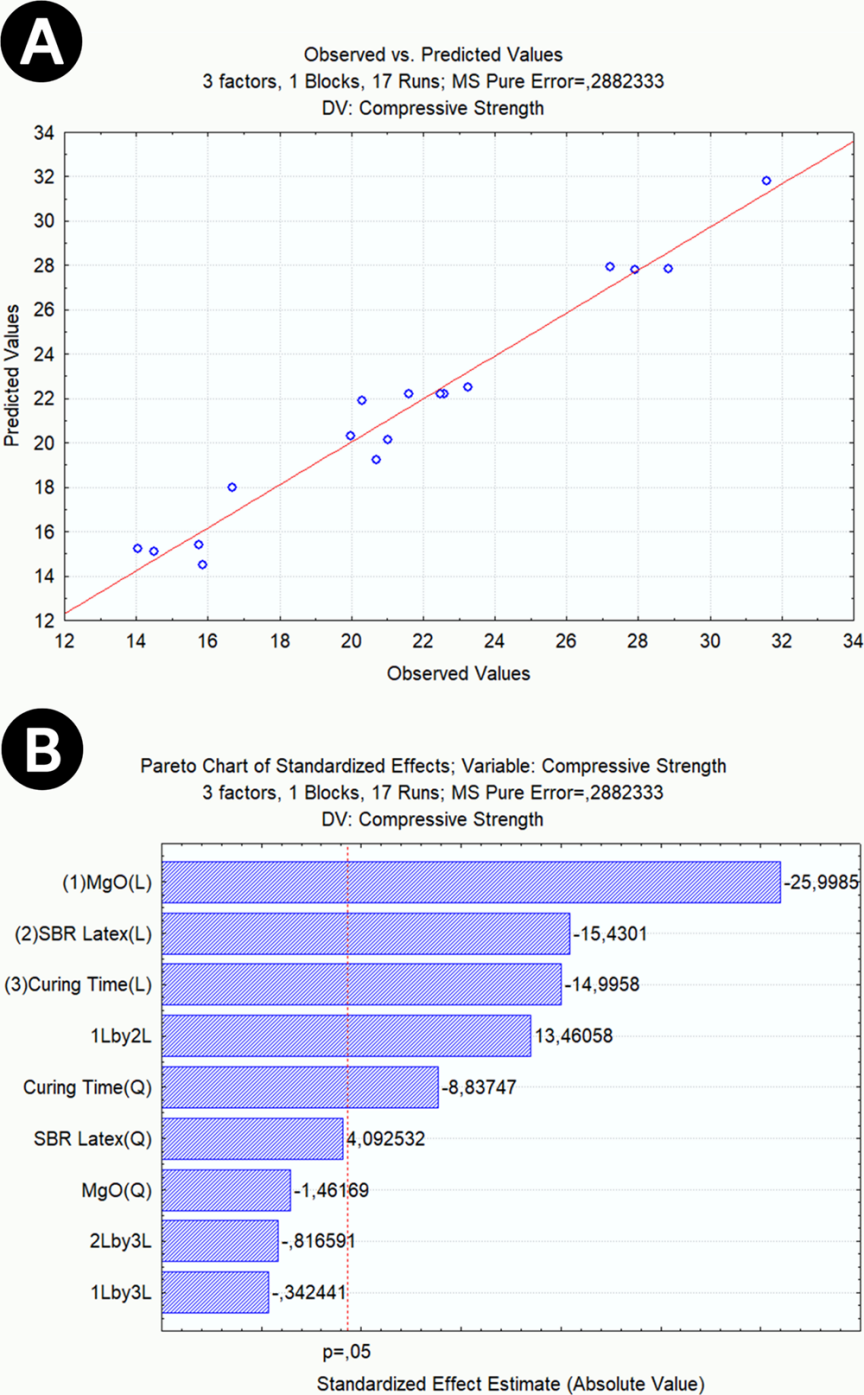
(A) Predicted values × observed values of the compressive strength analysis; (B) Pareto diagram of compressive strength analysis.

Figure 4A shows the predicted values versus observed values and demonstrates that the 17 tests carried out (blue dots) show coherent proximity to the line of predicted values from the quadratic model equation (red line). The residual values, the distance between the straight line and the points, are presented within a small dispersion. On average, there was a variation of 1.22% comparing each experimental value and its relative predicted by the model. Thus, it is possible to conclude that the model has normal behavior and a high level of agreement with the experimental results.

Figure 4B depicts the Pareto diagram and demonstrates the MgO variable as statistically significant for the linear interaction, the SBR Latex variable, and the curing time variables. As for the quadratic interaction, only the curing time variable demonstrated a significant impact. The variables MgO and SBR Latex showed a synergistic effect; that is, this behavior reveals that one additive is capable of affecting the influence of the other.

In this analysis scenario, MgO greatly influenced compressive strength more than the other variables. This is attributed to the way MgO acts in the cement structure.

The reduction in strength that occurs due to the increase in the amount of MgO at all ages of the experimental design is attributed to the lower formation of C–S–H as a result of the reduction in cement weight and its replacement by MgO.^36−41^

Researches examine the effects of MgO and C3A content on the properties of magnesium phosphate cement slurry, concluding that MgO significantly affects the strength and other properties of the cement slurry.^36−38^ The influence of MgO on the strength and microstructure of cement slurry has been highlighted, showing its impact on hydration characteristics at early ages of Portland cement slurry.^37,38^ This includes changes in microstructure and mechanical properties. The effects of magnesium oxide on the microstructure and mechanical properties of magnesium phosphate cement slurry are also significant, with notable implications for strength.^35^ Additionally, the influence of MgO on the rheology and compressive strength of cement slurries with high cement content, particularly for oil well applications, has been observed to cause significant changes in properties.^41,44^

Furthermore, MgO is a primarily expansive additive for cement slurries. However, the expansive effect can cause damage to the microstructure of the cement, such as cracks, and the increase in pore size and total volume, causing destruction in the microstructures of the interfaces.^47,48^ Therefore, expansion can harm mechanical strength and durability.^35^

#### 3.1.4 SEM Analysis

**Figure 5:**
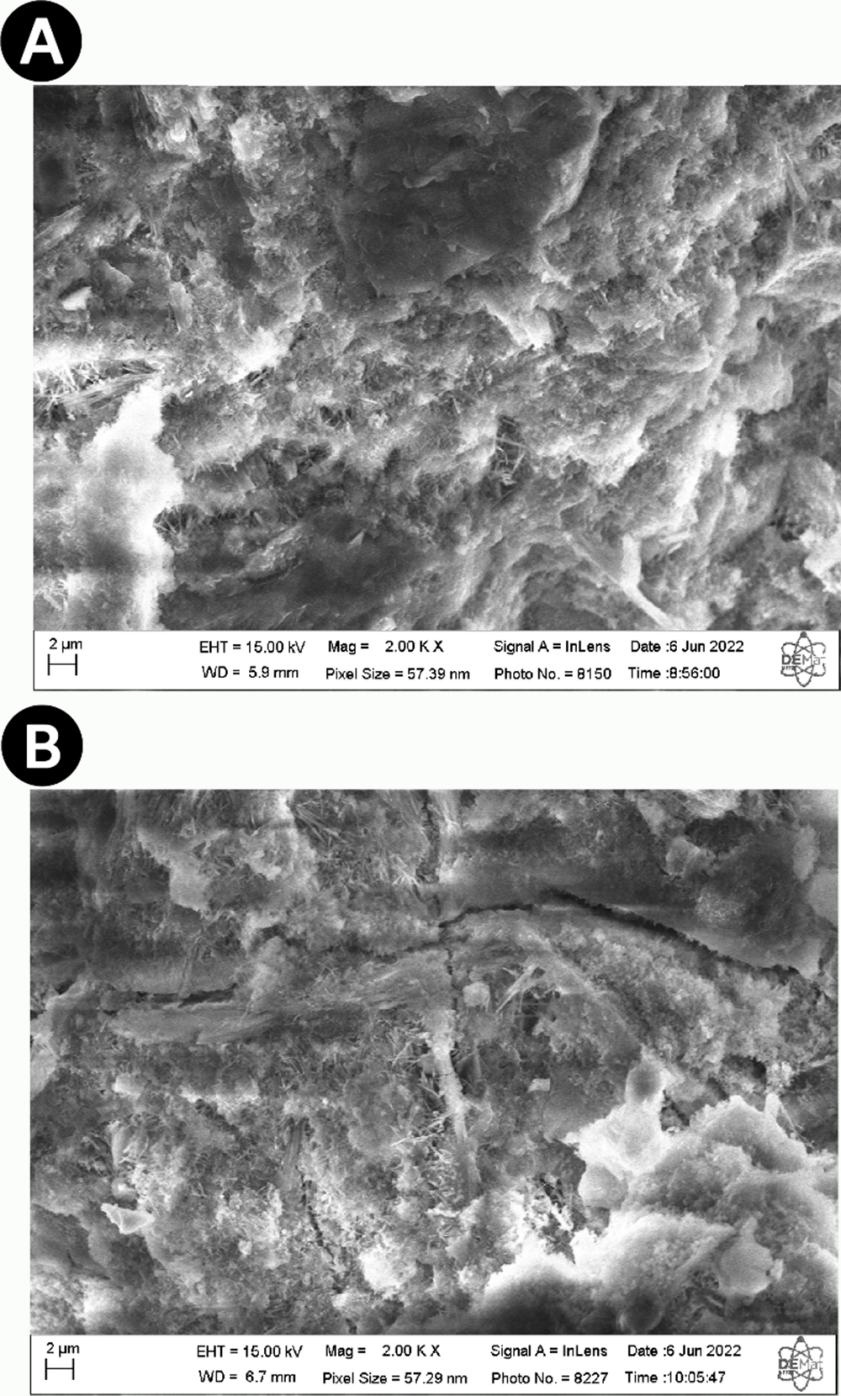
SEM images of cement slurries with formulations of 0.4% (A) and 3.2% (B) of MgO.

The result suggests that the expansion generated by MgO does not adequately contribute to the optimization of the cement microstructure. Even though it has a mechanism of action based on increasing particle size, decreasing pore size, and reducing total pore volumes, structuring from increased MgO can occur disorderly, promoting an increase in the number of failures and displacements in the microstructure. This situation, that is, the increase in the number of cracks and failures, can be observed through the SEM images in Figure 5, which compare images of cement slurries with formulations of 0.4% (A) and 3.2% (B) of MgO.

SBR Latex is characterized by a natural reduction in compressive strength by introducing elastic properties to the cement. This happens because SBR Latex is prone to hydrolysis in the alkaline environment of the cement composition and, therefore, causes excessive delay in the development of the cement’s compressive strength.^17^ This delay can be linked to the nonformation of Early Ettringite Formation (EEF) which, in turn, can generate Delayed Ettringite Formation (DEF).

**Figure 6:**
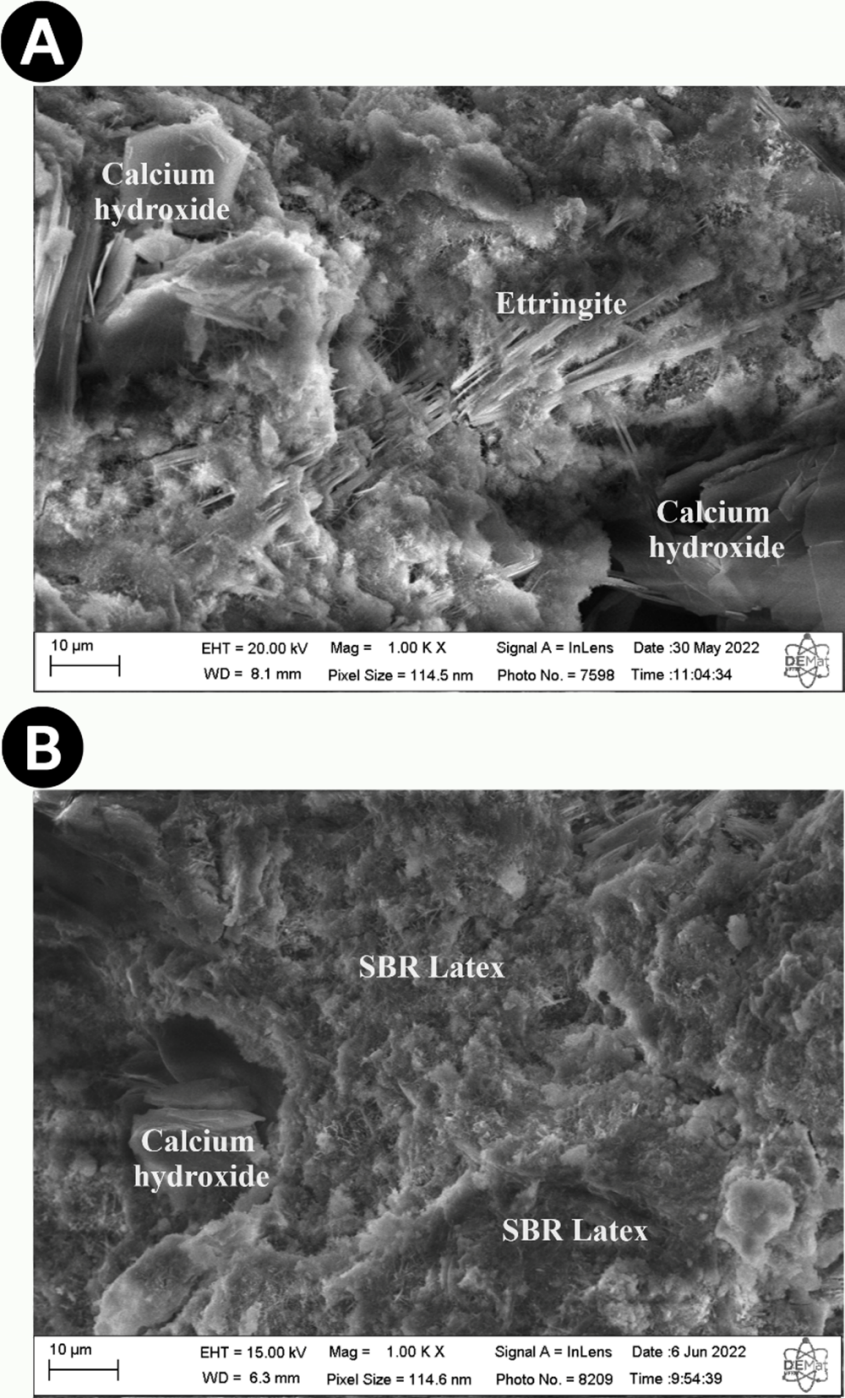
Samples of cement slurries with 0.4 gpc (A) and 1.6 gpc (B) of SBR latex.

Figure 6A depicts a Scanning Electron Microscopy (SEM) image analyzing a cement slurry containing 0.4 gpc of SBR latex with prominent presence of ettringite and calcium hydroxide. Ettringite, a crystalline phase of hydrated calcium sulfate, is clearly visible, displaying its characteristic structure. Additionally, calcium hydroxide, also known as hydrated lime, is observed in its crystalline form. These compounds are fundamental for the mechanical and chemical properties of the cement, contributing to its strength and durability. In Figure 6B, a cement slurry with a higher concentration of SBR latex, specifically 1.6 gpc, is examined. In this image, the presence of SBR latex is evident in various parts of the cement matrix. SBR latex is observed as small particles dispersed throughout the sample, suggesting a more homogeneous distribution of the additive in the cement. Moreover, the presence of calcium hydroxide is also identified, although in a lesser quantity compared to the previous image. This suggests that, with the increase in SBR latex concentration, the distribution and interaction of components in the cement slurry may be altered, affecting the characteristics of the final material.

As observed in this work, curing time is a variable inversely proportional to compressive strength. The quadratic effect indicates that the best compressive strength values are not entirely proportional to increased curing time in the MgO and SBR Latex system. The curing time variable reaches its ideal values with a lower value than the maximum established for the analysis variation.

The synergistic effect, that is, the result of one variable affecting the evelopment of the other, between the MgO and SBR Latex shows a positive impact on the compressive strength; however, both additives have an established action of reducing the compressive strength of cement slurries.

#### 3.1.5 Response Surfaces

**Figure 7:**
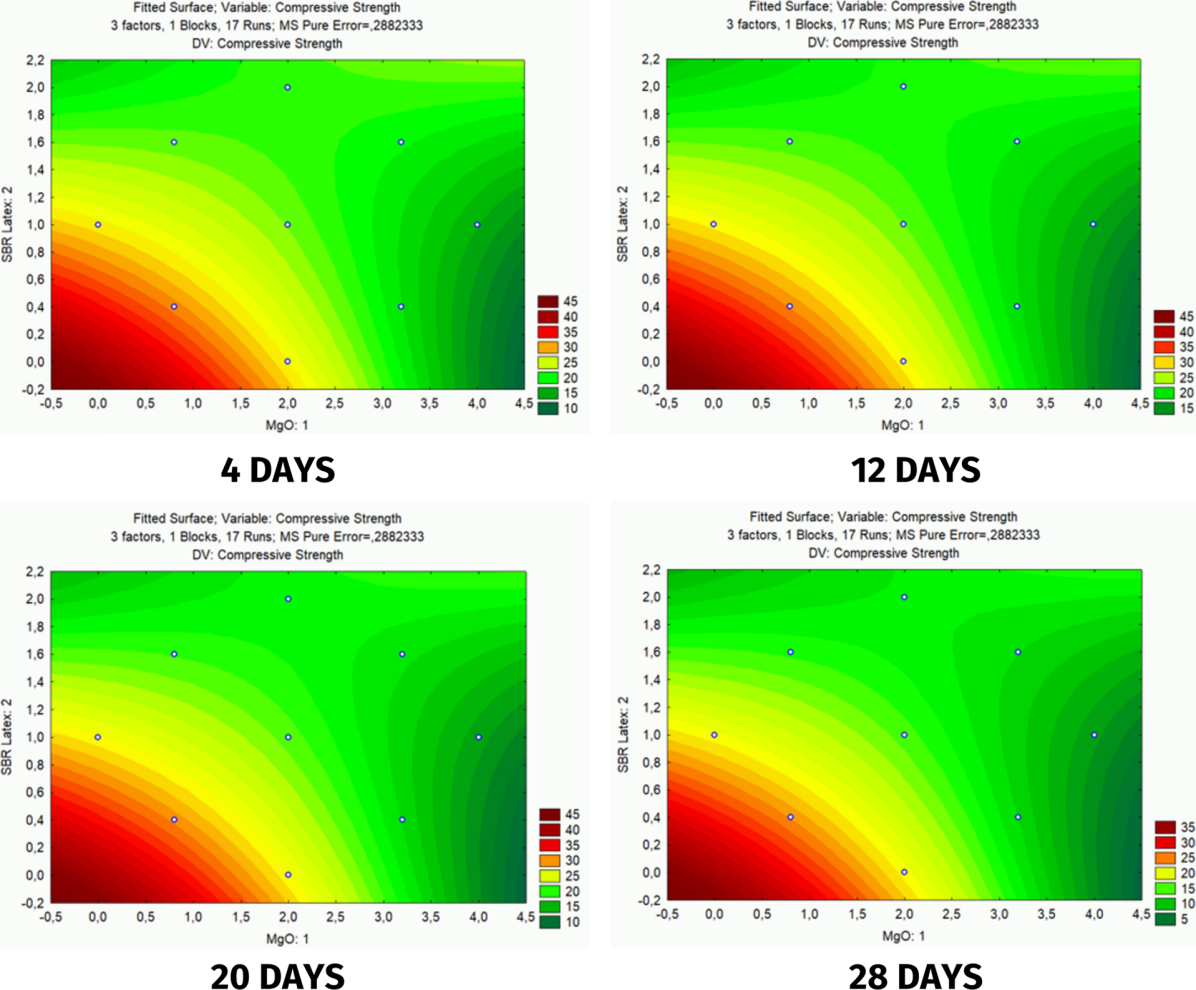
Response surfaces for the compressive strength analysis.

Figure 7 represents the relationship between the additives MgO and SBR Latex over the curing time of the slurry at ages of 4, 12, 20, and 28 days. This analysis is essential because it can observe how the two additives behave over time when combined.

The response surfaces in Figure 7 show that, in general, there was a slight variation in the performance of the additives over the observed range. However, the concentration of warm colors intensified in the lower left corner, suggesting that the two additives have a better synergistic effect when both are at low concentrations.

This pronounced synergistic effect at low concentrations may be associated with the mechanisms of action of the two additives, which resulted in positive responses. When MgO enters the hydration process, it generates microcracks in the system,^2^ which in turn are places conducive to the formation of DEF, and the latex can act on these cracks as a polymeric bridge, giving the system greater resistance.^40^

#### 3.1.6 Critical Values

The critical value coefficient is the value of the statistic that defines the upper and lower limits of a confidence interval or establishes the limit of statistical significance in a statistical test.

Considering the data in Table 7, the three factors presented critical values within the minimum and maximum observed ranges. This means that the ranges defined for each variable were adequate for the analysis; that is, if the critical values were close to the minimum or maximum values, this could be a suggestion that another study with more extensive ranges would be necessary.

**Table 7:** Critical Values of Compressive Strength Analysis

factor	minimum observed	critical value	maximum observed
MgO (%)	0	2.07	4
SBR latex (gpc)	0	1.88	2
curing time	4	9.88	28

The critical value of 9.88 for curing time explains the relationship between this factor’s linear interaction and the quadratic interaction, showing that it is unnecessary to reach observed extreme values to have an optimized response zone.

### 3.2 Shear Bond Strength Analyses (SBSA)

#### 3.2.1 Quadratic Model Equation

The SBSA results were subjected to the multivariate regression process, which aims to describe the relationships between the explanatory variables of the experiments. The regression showing a better fit with the results obtained was the quadratic regression.

Estimated effect coefficients describe the size and direction of the relationship between a term and the response variable. The effect for a factor represents the predicted change in response when the factor changes intensity. For example, how much the MgO factor can influence the shear strength response. The sign of the effect (positive or negative) indicates the direction of the relationship between the term and the response.

The second column of Table 8 groups all estimated effects for each factor involved as an independent variable and their correlations.

**Table 8:**
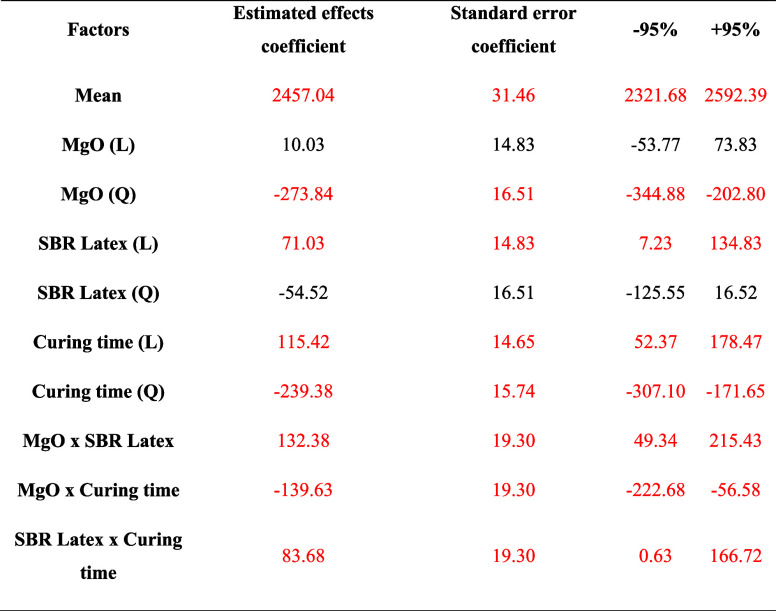
Shear Bond Strength Analysis Regression Coefficients

Only the statistically significant factors make up the quadratic model equation, highlighted in red in Table 8. Using the coefficients of the estimated effects, it is possible to perform a quadratic regression, which is a way of creating a model from an equation (eq 4):
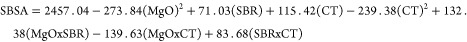
4

#### 3.2.2 Analysis of Variance: ANOVA

The analysis of variance (ANOVA) shown in Table 9 was conducted to evaluate the significance of the proposed model. The results indicate that the *F*-test value was 39.48, which is considerably higher than the critical *F* value from the table, which is 2.01. This result implies that the *F*-test value was 19.64 times greater than the tabulated *F* value, indicating strong evidence against the null hypothesis that all group means are equal, i.e., that the model is ineffective.

**Table 9:** ANOVA for the Quadratic Model^a^

source of variation	sum of square	degrees of freedom	mean square	*F* test
regression	1824122.00	9	202680.22	39.48
residual	35940.00	7	5134.29	
lack of fit	29979.00	5	5995.80	2.01
pure error	5961.00	2	2980.50	
total	1860062.00	16		

a *F*_0.05;9;7_ (*F*_table_) = 9.05.

The coefficient of determination (*R*^2^) was 0.981, suggesting that 98.1% of the variation in the response variable can be explained by the regression model. This *R*^2^ value indicates an excellent fit of the model to the observed data, demonstrating that the model equation captures almost all the variation in the response.

The F-test results, combined with the high *R*^2^, provide strong justification for the model’s viability. The significantly high *F*-test value suggests that there are statistically significant differences between the group means analyzed, while the high *R*^2^ value demonstrates that the model is highly effective in explaining the data variability.

The ANOVA conducted shows that the model is statistically significant and explains the majority of the observed variation, ensuring the quality and adequacy of the proposed model for describing the data.

#### 3.2.3 Observed and Predicted Values

**Figure 8:**
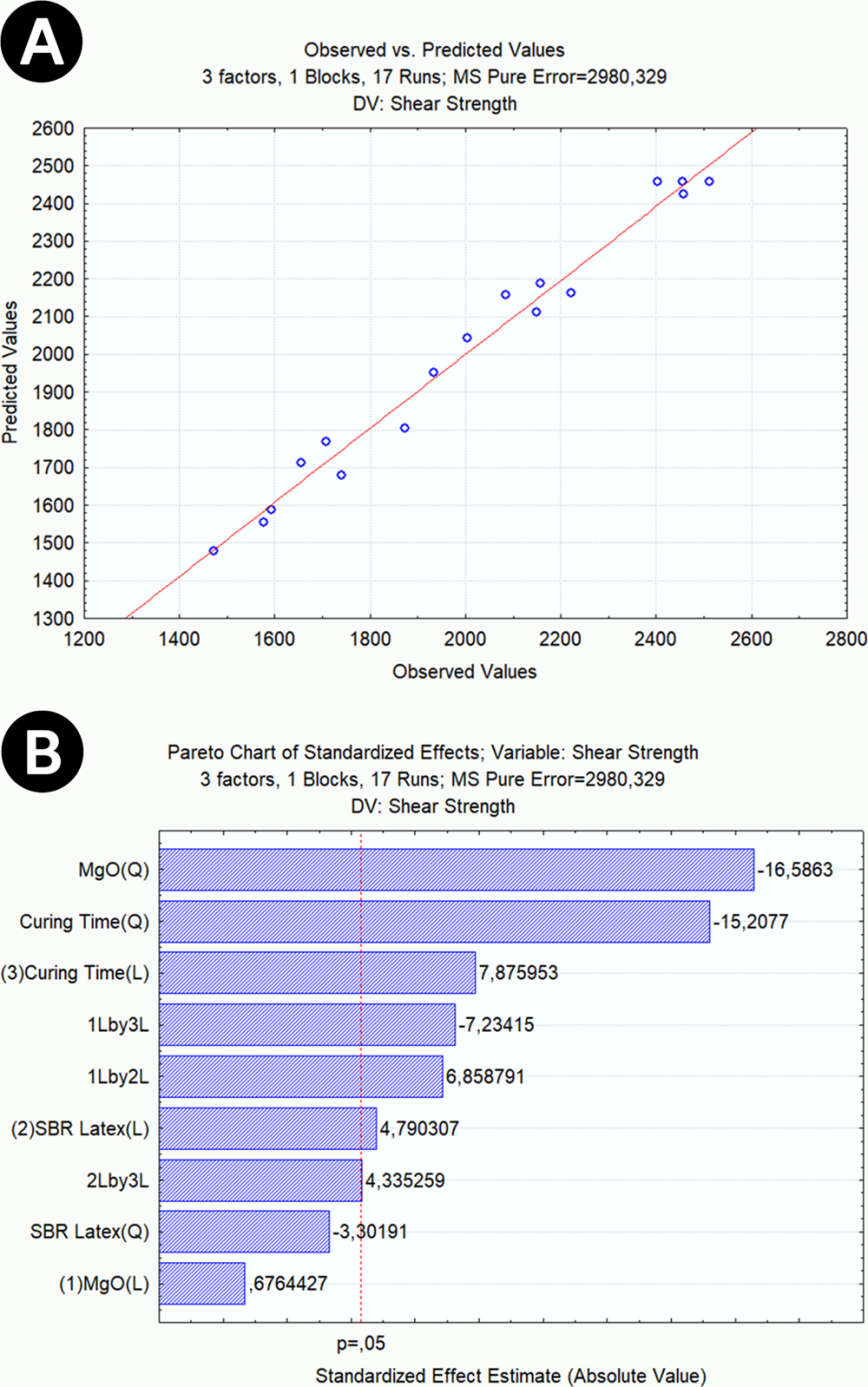
(A) Predicted values × observed values from Shear Bond Strength analysis; (B) Pareto Diagram for the adherence analysis.

Linear regression aims to quantify the relationship between the independent variables and the response variable. To do this, linear regression finds the line that best fits the data, known as the least-squares regression line. Figure 8A shows that this line produces a prediction for each observation in the data set, but typically, the prediction made by the regression line does not exactly match the observed value. This happens because the experimental procedure is exposed to many external factors that interfere with the observed value. However, there should not be high differences between predicted and observed values. The difference between those two values is called the residual. The Pareto diagram is used to determine the magnitude and importance of the effects of each factor. It shows the absolute values of the standardized effects from largest to smallest. The graph also plots a reference line to indicate which effects are statistically significant, where the bars that cross the reference line are statistically significant. Comparing the scattered representative points of the experiment and the least-squares regression line, an average variation of 2.32% was observed. This variation confirms that the model has normal behavior and a high level of agreement with the experimental results.

Figure 8B demonstrates which factors present effects of interaction that are significant for the constituted model and the response variable. The MgO factor showed the highest estimated effect value in the quadratic representation and practically none in the linear representation. When the quadratic term is significant, it indicates that a quadratic model can represent its response variable, and its response surface is no longer a plane. This result suggests that MgO acts in a remarkable and complex way. This behavior indicates that the MgO concentration can switch from a condition that contributes to the shear strength response to a situation in which it impairs the response.

Therefore, the amount of MgO in the cement must be controlled, as excess can cause cracks and failures in the material. This occurs because Mg(OH)_2_ is formed continuously and takes up more space than MgO. Thus, the hydration process for creating Mg(OH)_2_ from MgO generates an expansion force in the cement matrix that can cause excessive efforts and, consequently, failures. Therefore, it is concluded that although MgO is an additive promoting increased shear strength, excessive use can interfere negatively. In other words, MgO must be used in an ideal quantity so as not to compromise its positive performance.

MgO is widely used to compensate for drying shrinkage of cement-based materials to prevent cracking and loss of zonal isolation.^46^ The increase in MgO content in cement promotes more excellent production of Mg(OH)_2_, thus producing an expansive tension in the cement slurries, leading to more significant expansion.^41−43^

Different pressure and temperature stress types can affect the connection between the cement and the metallic coating. Expanding admixtures, such as MgO, can relieve this problem because they tend to expand after the initial setting, resulting in the development of a stressed condition in the cement that helps maintain the bond during pressure and temperature changes.^49−51^ With the appropriate expansion coefficient, crack formation is prevented, and existing cracks are filled. Subsequently, this ensures a good bond with the coating.^9,10,52^

Due to the high effect, the MgO variable also showed synergistic effects with the other variables, SBR Latex and curing time.

Cement hydration reactions occur over time, so curing time will always be an influential factor in the properties of the slurries. The variable curing time showed a statistically significant linear (7.875953) and quadratic (−15.2077) effect. Due to the fact that the linear effect is positive and the quadratic effect is negative, it is possible to infer that this variable initially provides a positive and later negative influence. This suggests that the most appropriate curing time is not near the minimum and maximum values observed.

Latex is an additive commonly used to control gas migration, fluid loss and improve the binding properties of cements. SBR latex is an aqueous dispersion of polymer copolymer made from butadiene, styrene, and unsaturated carboxylic acid through emulsion polymerization. It has high stability, compatibility with cement slurry, and good adhesion between oily and aqueous interfaces.^17,32^ Although it is usually used as an additive to control leaks, SBR latex is also capable of influencing other physical and chemical properties of cement slurries.^45^

The SBR Latex variable worked to improve the shear strength coefficient. This is because bonding agents such as latex enhance adhesion strength to metal piping. Including latex additives in the cement slurry reduces the surface tension between the slurry and the coating and helps the cement adhere to the coating. The surfactants present in latex can act on the coating surfaces, removing oil and allowing better adhesion contact.^53^

Nakayama and Beaudoin was analyzed the shear strength of cement slurries for oil wells with six different types of latex.^15^ It was found that, in general, the bond strength is improved with the addition of latex, as this addition generally increases the adhesion of the cement slurry to the steel. It also found that the maximum bond strength of latex-modified cement was typically achieved within about 24 h of hydration. This information, combined with experimental results, indicates that SBR latex acts more strongly in the early curing ages of the cement slurry.

#### 3.2.4 Response Surfaces

**Figure 9:**
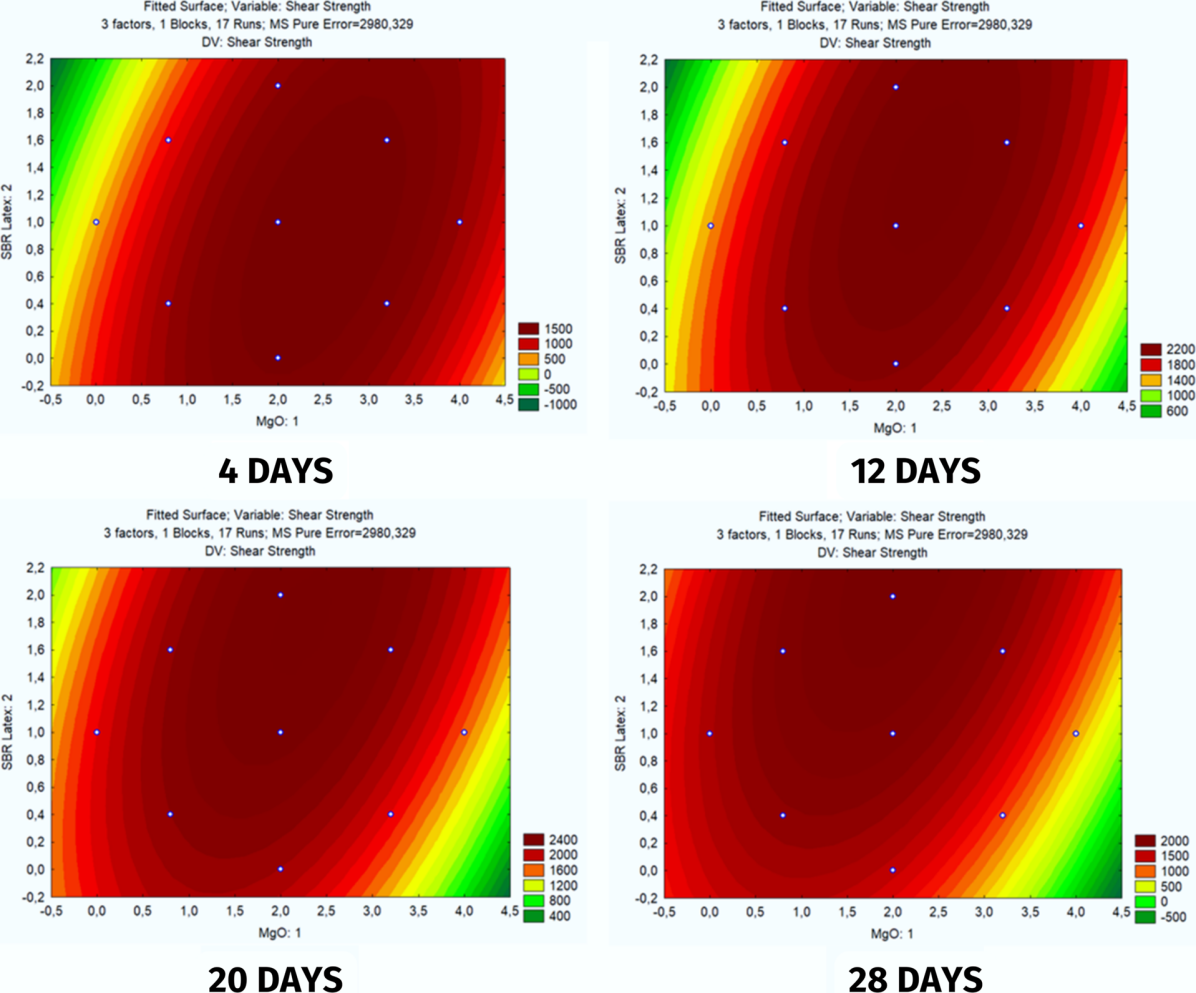
Shear Bond Strength analysis response surfaces.

Figure 9 represents the relationship between the additives MgO and SBR Latex over the curing time of the slurry at ages of 4, 12, 20, and 28 days. By comparing the images, it is possible to evaluate how the MgO and SBR Latex additives affect the shear strength result.

It is noted that after 4 days of curing, the concentrations of MgO and Latex that best provide shear strength to the cement slurry are in a zone close to the average values of both additives. This behavior changes over time, demonstrating a vertical displacement of the optimized zone. This behavior indicates that SBR Latex becomes an increasingly less efficient variable over time.

The optimized zones close to the center of the response surfaces demonstrate that the variations used in the factors are valid and that the information found is more accurate.

#### 3.2.5 Critical Values

Critical values are, essentially, values that define the zones where each factor had an optimized influence on the response variable. These values corroborate the conclusions made during the Pareto Diagram and response surface analyses. Table 10 shows the minimum, critical, and maximum observed values.

**Table 10:** Shear Bond Strength Analysis Critical Values

factor	minimum observed	critical value	maximum observed
MgO (%)	0	2.25250	4
SBR latex (gpc)	0	1.72405	2
curing time	4	18.73441	28

All analyzed variables presented a critical value within the minimum and maximum observed interval. This is in line with previous analyses.

The variables MgO and curing time are the most likely variables that should influence shear strength in a controlled manner. Excessive increases in these variables can compromise the adhesion of cement slurries. The SBR Latex variable demonstrates a more discrete influence. From the model generated, it is possible to identify that this factor can offer more significant effects at higher concentrations.

## 4 Conclusions

This work analyzed various properties of cement slurries for oil wells subject to the influence of variables such as MgO concentration, SBR Latex concentration, and curing time. Analyzes were developed focusing on the additive in the performance of MgO and SBR Latex, separately and simultaneously. From the results discussed, it is concluded that

- Considering the compressive strength developed in the first 24 h of curing under well conditions, the MgO additive has no effect. Acting only in the most advanced phases of slurry hydration.
- For the compressive strength developed between the ages of 4 and 28 days, MgO, through its expansive property, does not adequately contribute to optimizing the cement microstructure.
- For the, the presence of SBR Latex reduces the value of this property due to the retardant effect generated by the chemical reactions of SBR Latex, which hinders the formation of ettringite and increases the maturity of the slurry and the amount of water in the microstructure.
- Concerning the compressive strength developed between the ages of 4 and 28 days, the combined use of MgO and SBR Latex favors the compressive strength of these cementitious materials.
- Even though MgO is an additive that commonly promotes increased shear strength, excessive use can interfere negatively. Therefore, MgO must be used in ideal quantities to avoid compromising its positive performance.
- In the analyzed scenario, the curing time positively influenced the shear strength of cement slurries in the first ages and negatively in the following ages. This suggests that as the well ages, there is a decrease in shear strength.
- Latex is a variable that has a more discrete influence on the shear strength of cement slurries. The model generated also indicates that SBR Latex becomes an increasingly less efficient variable over time.
- The combined use of MgO and SBR Latex in cement slurries for oil wells generates a positive synergistic effect on the mechanical properties of compressive strength and shear strength. In other words, these additives can be present in the same formulation, performing their functions and promoting contributions between both.

The analysis described in this work highlights the critical conclusion that achieving all the expected objectives using only one additive is impossible. All additives have characteristics that may be suitable for one situation and not for another, so it is worth highlighting this in addition to dealing with additives primary and secondary effects.
